# Phase Formation during Solidification of Mg-Nd-Zn Alloys: An In Situ Synchrotron Radiation Diffraction Study

**DOI:** 10.3390/ma11091637

**Published:** 2018-09-06

**Authors:** Domonkos Tolnai, Tungky Subroto, Serge Gavras, Ricardo Buzolin, Andreas Stark, Norbert Schell, Norbert Hort

**Affiliations:** 1 Institute of Materials Research, Helmholtz-Zentrum Geesthacht, Max-Planck-Strasse 1, D 21502 Geesthacht, Germany; tungky.subroto@brunel.ac.uk (T.S.); sarkis.gavras@hzg.de (S.G.); ricardo.buzolin@tugraz.at (R.B.); andreas.stark@hzg.de (A.S.); norbert.schell@hzg.de (N.S.); norbert.hort@hzg.de (N.H.); 2 Brunel Centre for Advanced Solidification Technology, Brunel University London, London UB8 3PH, UK; 3 Institute of Materials Science, Joining and Forming, Graz University of Technology, Graz A8010, Austria

**Keywords:** magnesium, Mg-Nd-Zn alloys, in situ solidification, synchrotron radiation diffraction

## Abstract

Mg-4Nd base alloys with Zn additions of 3, 5 and 8 wt % were investigated with in situ synchrotron radiation diffraction during solidification. This method enabled the investigation of phase formation and transformation in the alloys. The diffraction results were supported with TEM observations on the as-solidified samples. The results show the effect of increased Zn addition on stabilizing the Mg_3_RE phase (RE—rare earth). The experimental results agree only partially with the theoretical calculations indicating the need to improve the existing thermodynamic database on the alloy system.

## 1. Introduction

The increased demand for “green” solutions and eco-friendly vehicles has drawn attention to lightweight materials. Mg, as a metal with outstanding specific properties [1], is a promising candidate to replace heavier counterparts. With conscious alloying, the poor absolute properties and corrosion resistance of these materials can be improved significantly [2]. Furthermore, Mg is being used as a degradable implant material in the medical sector [3], where its suitable mechanical properties and controlled corrosion play an important role.

The addition of Zn to Mg as an alloying element results in the simultaneous increase in strength and ductility [4]. This effect made ZK60 (Mg-6.0Zn-0.6Zr (wt %)) one of the high strength commercial wrought Mg alloys. The significantly enhanced properties of the Mg-Zn system compared to Mg microalloyed with Ca or RE (rare earth) has been a motivation to further improve its mechanical property profile via the engineering of grain boundary phases [5,6,7,8,9]. These investigations report enhancement of strength and ductility [10,11] by the addition of different RE elements to the ZK system [12]. Neodymium, with its low solid solubility (3.6 wt % at 549 °C [13]), is an ideal element because relatively low concentrations are necessary to introduce secondary phase particles that further improve strength at elevated temperatures [14,15]. Due to the low cytotoxicity of Nd, the Mg-Nd-Zn system is also considered as a prospective alloy for bio-absorbable implants [16].

In situ diffraction experiments during solidification reveal the phase-formation and transformations as a function of temperature and cooling rate [17]. Identifying the secondary phases and determining their solidification sequence contribute to the validation of existing phase diagrams and to the development of more accurate thermodynamic databases [18]. Due to the relatively large sample size, the use of neutron sources provides good statistics compared to synchrotron sources [19]. However, the latter yields an excellent time-temperature resolution [20], allowing a more realistic model of casting conditions during the experiments to be created.

The aim of this research is to investigate the phase relation during solidification in Mg-Nd-Zn alloys with synchrotron radiation diffraction and to elucidate the effect of varying Zn additions with regard to microstructure and mechanical properties.

## 2. Materials and Methods

The alloys investigated were cast by permanent mold indirect chill casting [21]. Pure Mg was melted in an electric resistance furnace under a protective atmosphere with a mixture of Ar and 2 vol % SF_6_. The Nd and Zn were added in pure form, whilst the melt was continuously stirred. The melt was held at 750 °C for 10 min, then poured into a preheated steel mold at 660 °C. The crucible with the molten alloy was held at this temperature for 15 min and subsequently immersed into a room temperature water bath at a rate of 10 mm/s.

Four alloy compositions were investigated in this work: Mg-4Nd, Mg-4Nd-3Zn, Mg-4Nd-5Zn and Mg-4Nd-8Zn. All alloy compositions described in this work are stated in wt %, unless stated otherwise. The actual compositions of the alloys were measured with a Spectro Spectrolab M9, (Kleve, Germany) spark analyzer. The Nd content was also measured using a Bruker S5 (Billarica, MA, USA) X-ray fluorescence (XRF) to determine the exact amounts of Nd in the investigated alloys.

The thermal properties of the alloys during melting and solidification were analyzed by a Differential Thermal Analyzer (DTA) using a MettlerToledo TGA/SDTA 851e (Colombus, OH, USA) under an Ar atmosphere. The samples were contained in a Mettler Toledo stainless steel crucibles (ME 29990) with a diameter of 6 mm, height of 5.5 mm and a wall thickness of 0.5 mm, and were heated and cooled in the range of 250 °C–700 °C. The heating and cooling rates were set to 10 °C/min and an empty crucible was used as a reference. The heating-cooling cycles were repeated three times to ensure reproducibility. The thermodynamic simulations were performed with the software PandaT using the PanMg2017 database.

The in situ synchrotron radiation diffraction experiments were performed at the P07 (HEMS) beamline of PETRA III. DESY (Deutsches Elektronen-Synchrotron, Hamburg, Germany) A monochromatic beam was used with an energy of 100 keV (λ = 0.0124 nm) and a cross-section of 1.1 × 1.1 mm. The acquisition time of each diffraction pattern was set to 0.5 s. The samples were enclosed in Mettler Toledo stainless steel crucibles (ME 29990) under an Ar atmosphere. The experiments were performed in the chamber of a modified DIL 805 A/D (TA Instruments, New Castle, DE, USA) dilatometer. The sides of the chamber were covered with Kapton windows and the coil was modified in order for the beam to pass through the sample only. The diffraction patterns (Debye–Scherrer rings) were recorded by a Perkin Elmer 1621 Flatpanel detector (Waltham, MA, USA), with an effective pixel size of (200 μm)^2^, placed 1603 mm behind the sample, calibrated by a LaB_6_ standard powder sample. The samples were heated at a constant rate of 50 and 100 K/min up to 800 °C, then held for 5 min to ensure temperature homogeneity and subsequently cooled with the same rate to 200 °C. The diffraction patterns were integrated azimuthally and analyzed with the software Fit2DESRF, Grenoble, France. Line profiles were obtained by integrating the diffraction patterns in the azimuthal direction.

Specimens for scanning electron microscopy (SEM) analysis were prepared by grinding and polishing using SiC paper and OPS solution, respectively. A TESCAN Vega SB-U III SEM Brno, Czech Republic with an accelerating voltage of 15 kV and working distance of 15 mm was used for microstructural characterization. The area fraction of intermetallic compounds was measured from a minimum of 10 randomly distributed regions of a specimen captured by backscattered electron (BSE) micrographs and analyzed using ImageJ software NIH, Bethesda, MA, USA.

A Philips CM200 (Amsterdam, the Netherlands) transmission electron microscope (TEM) equipped with an Energy Dispersive X-ray Spectrometer (EDXS) (Oxford instrument, Oxfordshire, UK) was used for characterization at 200 kV accelerating voltage. The bright-field mode was used for imaging the microstructure while the diffraction pattern information from the intermetallic particles was obtained through selected area electron diffraction (SAED). The TEM foils were prepared by Focused Ion Beam milling (FIB) using a FEI Nanolab200 Dual Beam Scanning Electron Microscope (Hillsboro, OR, USA). CaRIne crystallographic software (Version 3.1) was used to calculate the theoretical diffraction patterns. The results from CaRIne simulations and the Pearson’s crystallographic database (PCD) (Rel. 2015/2016) were used to carry out phase identification.

## 3. Results

The chemical composition of the cast ingots of the alloys was measured by X-Ray fluorescence spectroscopy (XRF) and spark analysis (Table 1).

**Figure 1:**
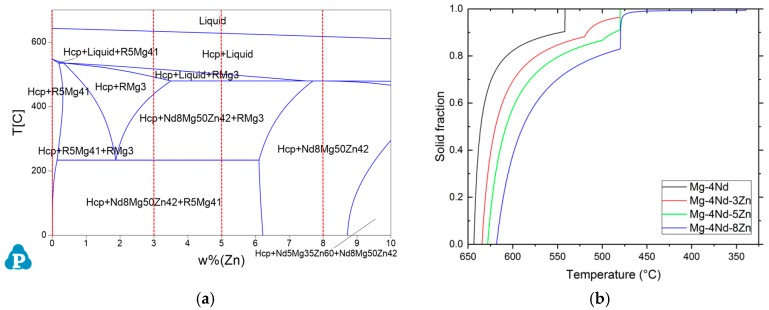
(**a**) Pseudo-binary diagram for the Mg-4Nd-*x*Zn (*x* = 0 to 10 wt %) system calculated using PandaT. Red dashed lines represent the alloy compositions used in this work; Zn = 0, 3, 5 and 8 wt %; (**b**) Scheil solidification calculations.

In order to investigate the phase compositions of the alloys and to obtain the phase formation temperatures the pseudo-binary phase diagram of the Mg-4Nd-xZn system has been constructed with Scheil cooling calculations using PandaT (Figure 1).

The binary Mg-4Nd alloy consists of -Mg and Mg_41_Nd_5_ intermetallics. The ternary alloys with the lower Zn concentrations in addition to these two phases consist of Mg_50_Zn_42_Nd_8_, which is known as the T2 phase [22]. The highest Zn containing alloy (Mg-4Nd-8Zn) only has this intermetallic and -Mg present in its microstructure. In all the alloys investigated, the solidification starts with the formation of -Mg dendrites. The primary Mg dendrites form at 643 °C in the binary alloys, 634 °C in Mg-4Nd-3Zn, 628 °C in Mg-4Nd-5Zn and 622 °C in Mg-4Nd-8Zn. The binary alloy reaches the eutectic point at 542 °C where the remaining liquid solidifies as -Mg/Mg_41_Nd_5_ eutectic. In the Mg-4Nd-3Zn and -5Zn alloys, the formation of -Mg is followed by the solidification of the Mg_3_Nd phase at 520 °C and 500 °C, respectively. The solidification ends in both instances with the formation of Mg_50_Zn_42_Nd_8_ at 480 °C. In the highest Zn containing alloy, Mg-4Nd-8Zn, the Mg_50_Zn_42_Nd_8_ phase forms after the primary -Mg at 480 °C. The solidification ends at 340 °C.

**Figure 2:**
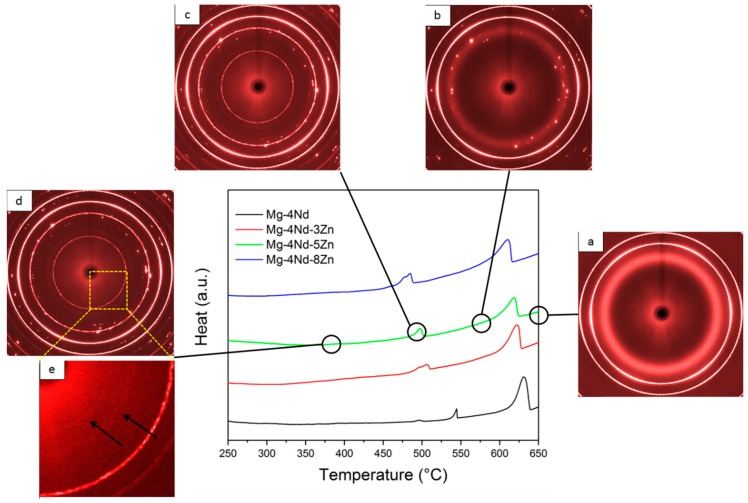
DTA curves of the samples obtained during solidification and the corresponding diffraction patterns of Mg-4Nd-5Zn in (**a**) fully liquid; (**b**,**c**) semi solid and (**d**,**e**) fully solid state, obtained by in situ synchrotron radiation diffraction.

The DTA curves of the investigated alloys obtained during solidification are shown in Figure 2, along with the diffraction patterns acquired by in situ synchrotron radiation diffraction at the different stages of solidification.

The DTA investigations show that the solidification of -Mg dendrites starts in the binary alloy at 639 °C. In the ternary alloy with 3 wt % Zn the primary Mg solidifies at 629 °C, in the 5 wt % Zn containing alloy at 625 °C and in the 8 wt % Zn containing alloy at 617 °C. The formation of the eutectic in the binary alloy occurs at 545 °C, while the formation of the intermetallic phase in Mg-4Nd-3Zn occurs at 510 °C, in Mg-4Nd-5Zn at 502 °C and in Mg-4Nd-8Zn at 490 °C, respectively. In the liquid phase, the diffraction rings originate from the steel crucible holding the melt. The diffuse ring is caused by the portion of the alloy that is still in the molten state. In the background of the diffuse ring, diffraction spots appear as the cooling proceeds and -Mg starts to solidify. At the temperatures of the intermetallic phase, the formation of the diffraction peaks of the intermetallic phases complement the ones from the -Mg. As there is no more molten alloy in the system, the diffuse ring disappears during solidification.

**Figure 3:**
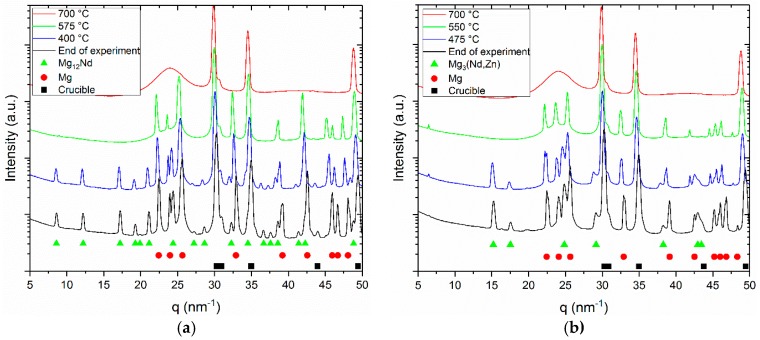

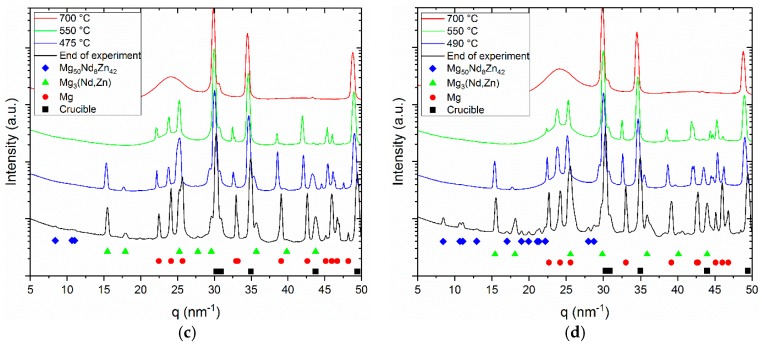
Line profiles obtained by the azimuthal integration of the diffraction patterns acquired during the in situ solidification experiments with 50 K/min cooling of (**a**) Mg-4Nd; (**b**) Mg-4Nd-3Zn; (**c**) Mg-4Nd-5Zn and (**d**) Mg-4Nd-8Zn.

The azimuthally integrated line profiles are shown in Figure 3a–d.

The red profiles represent the liquid state. The hump originates from the amorphous background signal, while the crystalline peaks are diffracted from the steel crucibles. In the second profiles (green) besides the crucibles, peaks can be observed from the primary Mg dendrites. In the third profiles (blue), these are complemented by the peaks of the intermetallic phases. The last profiles are integrated from the Debye-Scherrer patterns acquired at the end of the experiment at 200 °C. The peaks move towards higher q values because of the decrease in lattice parameter while cooling down.

Lattice parameters for Mg_3_(Nd, Zn) and Mg_50_Nd_8_Zn_42_ were extracted by solving interplanar spacing equations of its corresponding crystal structure by fitting the diffraction peak in the line profiles using a Pseudo Voigt function. The lattice parameters were calculated based on the determined peak positions. The evaluations were carried out on the line profiles of the ternary alloys cooled at 100 °C/min at the end of experiment (~50 °C). The line profiles of Mg-4Nd-3Zn, Mg-4Nd-5Zn and Mg-4Nd-8Zn were used to extract the lattice parameters for the quasi-binary Mg_3_ (Nd, Zn) intermetallic phase. (111) and (200) peaks were used to determine the exact value. These peaks were chosen due to their fitting feasibility. The interplanar spacing equations for cubic crystals were used to obtain lattice parameters of this phase. The resulting value (a = b = c) for Mg-4Nd-3Zn is 0.716 nm, Mg-4Nd-5Zn is 0.703 nm, and Mg-4Nd-8Zn is 0.696 nm. The obtained value for each composition is the mean of the lattice parameters from two peaks.

A c-center orthorhombic interplanar spacing equation was also used to obtain the lattice parameters of the Mg_50_Nd_8_Zn_42_ intermetallic phase. The lattice parameters for the Mg_50_Nd_8_Zn_42_ phase were only calculated for Mg-4Nd-5Zn and Mg-4Nd-8Zn alloys, as this intermetallic phase was not observed in the Mg-4Nd-3Zn alloy. From the Mg_50_Nd_8_Zn_42_ phase, (110), (020) and (003) peaks were used to determine the lattice parameters. The obtained lattice parameters for different alloys are as follows: Mg-4Nd-5Zn (a = 0.992, b = 1.139 and c = 0.948) and Mg-4Nd-8Zn (a = 0.983 nm, b = 1.134 nm, c = 0.943 nm). The (020) peak on Mg-4Nd-5Zn could not be fitted due to its geometry, thus the peak position from the line profile was directly used for calculation. The lattice parameter calculation of Mg_50_Nd_8_Zn_42_ was performed using peaks with a low scattering vector magnitude (low q value, i.e., q < 25 nm^−1^). This phase was simulated in CaRIne using a primitive C-center orthorhombic structure, thus at a higher q value, i.e., q > 25 nm^−1^, there are narrowly spaced peaks with low intensity. This could lead to a higher indetermination error of the peak position, thus increasing the error of the lattice parameter analysis.

**Figure 4:**
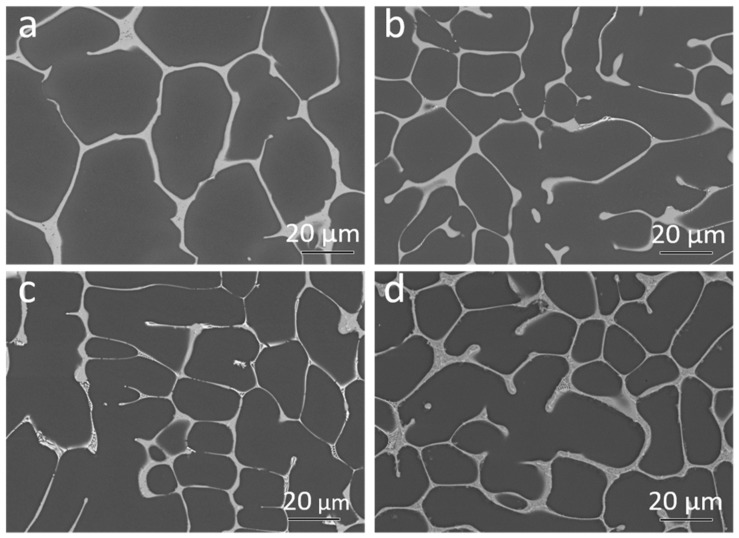
Back Scattered Electron micrographs of the samples after solidification of (**a**) Mg-4Nd; (**b**) Mg-4Nd-3Zn; (**c**) Mg-4Nd-5Zn and (**d**) Mg-4Nd-8Zn.

The Back Scattered Electron (BSE) micrographs of the samples after solidification are shown in Figure 4a–d.

**Figure 5:**
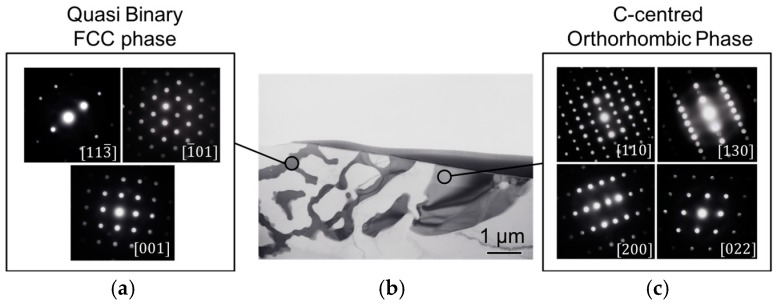
TEM observation of Mg-4Nd-5Zn in Selected Area Electron Diffraction (**a**) and (**c**) of two distinct intermetallic phases; (**b**) bright field mode.

The area fraction of intermetallic particles was calculated based on the BSE micrographs. The binary Mg-4Nd exhibited 10.8 ± 1.2% intermetallics while the ternary alloys Mg-4Nd-3Zn, Mg-4Nd-5Zn and Mg-4Nd-8Zn contained 13.9 ± 1.7%, 13.4 ± 0.4%, and 15.2 ± 0.7%, respectively. The addition of Zn to Mg-4Nd increased the intermetallic area fraction and accordingly the Mg-4Nd-8Zn exhibited the highest area fraction. A semi-continuous network of intermetallic particles along the grain boundary was observed for the investigated alloys. The Mg_12_Nd intermetallic compound shows a continuous morphology as observed in Figure 4a for the Mg-4Nd alloy. In the ternary alloys two different morphologies of the intermetallic compounds were observed; a continuous intermetallic compound and a lamellar one. The crystal structures of the intermetallic phases were identified by transmission electron microscopy as shown in Figure 5.

Figure 5b shows a bright-field TEM image of an area with two different intermetallic morphologies in the Mg-4Nd-5Zn alloy. Microbeam diffraction analysis performed on each intermetallic morphology was used to confirm the intermetallic phases present. The intermetallic compound with lamellar morphology (Figure 5a) was identified as having an FCC (face centered cubic) crystal structure corresponding to the quasi-binary Mg_3_(Nd, Zn) intermetallic phase [23]. The continuous-like intermetallic compound (Figure 5c) was indexed according to a C-centered orthorhombic crystal structure and it corresponds to the Mg_50_Nd_8_Zn_42_ intermetallic phase [22]. Both phases were also detected and characterized in the Mg-4Nd-8Zn alloy as reported previously [24].

## 4. Discussion

The results of the thermodynamic calculations in equilibrium partially support the experimental results. In the case of the binary Mg-4Nd alloy, the results are in accordance with the findings of Easton et al. [25]. However, this equilibrium phase can only be reached after long- term thermal treatment. Under realistic casting conditions, the Mg_12_Nd phase is present in the alloy. In regard to the ternary alloys, the results show some inconsistencies with the experimental findings. In the Mg-4Nd-3Zn alloy, the predictions show the presence of two intermetallic phases: Mg_3_(Nd, Zn) and Mg_50_Nd_8_Zn_42_. Although both phases could be found on the SEM micrographs, the volume fraction of Mg_50_Nd_8_Zn_42_ was too low to determine their presence with synchrotron radiation diffraction. This indicates that the alloy composition lies on the border of phase fields in the phase diagram. Therefore, the segregation and the resulting minor inhomogeneities can be responsible for the rare occurrence of the latter intermetallic. Another inaccuracy of the calculations compared to the experimental result is in the case of the highest Zn containing alloy, Mg-4Nd-8Zn. The equilibrium calculations predict the presence of only one intermetallic (Mg_50_Nd_8_Zn_42_) in the fully solid state. However, the experiments reveal the presence of the other intermetallic, Mg_3_(Nd, Zn) [24]. As expected, the Scheil calculations are closer to the observed results; however, in the case of the lower Zn containing alloys, the equilibrium calculation indicates a solid state transformation of Mg_3_(Nd, Zn) to the tetragonal Mg_12_Nd, which did not take place in the experiments. In Mg-Nd alloys, the Mg_41_Nd_5_ tetragonal phase is considered to be the stable phase preceded by the Mg_3_Nd and Mg_12_Nd phases in this order. The results suggest that the presence of Zn stabilizes the most unstable Mg_3_Nd phase under realistic solidification conditions as well.

The increase of Zn addition shifts the diffraction peaks of the intermetallic phases to higher diffraction angles. This indicates a reduction in the lattice parameters as found in other investigations [23,26]. This can be attributed to the smaller atomic radius of Zn (0.134 nm), compared to Nd and Mg (0.16 and 0.181 nm, respectively). The lattice parameters obtained in this work are comparable to the value that was determined in previous investigations [22,23]. The lattice parameters of the ternary intermetallic phase (C-center orthorhombic) were found to be: a = 0.965–0.984 nm, b = 1.118–1.135 nm and c = 0.946–0.963 nm [22]. For the quasi-binary intermetallic phase (FCC), a = b = c = 0.68–0.74 nm was found [22,23].

No notable effect of the investigated cooling rates on the solidification sequence was observed in the investigated alloys. Phases were formed at higher temperatures when samples were cooled faster −100 °C/min, as compared to the slower cooling rate −50 °C/min. Although Khan [27] observed a similar trend on different Mg-alloys, this result is unexpected. Usually, higher cooling rates correspond to higher undercooling. However, previous works on in situ solidification using synchrotron radiation diffraction for Mg-RE alloys reported that the relationship between phase formation temperatures with cooling rate is not clear [28]. This suggests there might be a slight shift between the measured temperature with the sample temperature inside the crucible, and such a shift is a function of the chosen cooling rate. Moreover, the time-temperature resolution for the experiments carried out using the cooling/heating rate of 50 °C/min is higher compared to 100 °C/min experiments; thus, it is possible that a deviation occurs in the temperature measurement.

## 5. Conclusions

The solidification properties of Mg-4Nd-xZn alloys have been investigated with in situ synchrotron radiation diffraction. From the findings, the following conclusions can be drawn:

- The solidification sequence of the binary Mg-4Nd alloy starts with the formation of the -Mg dendrites followed by the eutectic solidification of -Mg/Mg_12_Nd.
- In the Mg-4Nd-3Zn alloy, the -Mg is followed by the solidification of Mg_3_(Nd, Zn) during cooling.
- In the case of the high Zn containing alloys, these phases are complemented by Mg_50_Nd_8_Zn_42_.
- In comparison with the equilibrium phase diagram calculations, some differences can be found. Under realistic casting conditions, the intermetallic phase in Mg-4Nd is the Mg_12_Nd instead of the stable Mg_41_Nd_5_.
- The addition of Zn stabilizes the Mg_3_(Nd, Zn) phase, which is meta-stable in the binary Mg-Nd alloys. Furthermore, it promotes the formation of the Mg_50_Nd_8_Zn_42_ phase.
- The lattice parameters of these intermetallic phases decrease with the addition of Zn, in accordance with the smaller atomic radius of Zn.
- Applying cooling rates of 100 °C/min and 50 °C/min did not induce remarkable changes in the formation temperatures, and no changes in the intermetallic stoichiometries.

## Figures and Tables

**Table 1:** Chemical composition of the investigated samples.

Nominal Composition	Nd wt % (Spark)	Nd wt % (XRF)	Zn wt % (Spark)
Mg-4Nd	>4.20	4.40	trace
Mg-4Nd-3Zn	>4.20	4.35	3.20
Mg-4Nd-5Zn	>4.20	4.20	5.20
Mg-4Nd-8Zn	>4.20	4.34	8.00
